# Hijacking of the Pleiotropic Cytokine Interferon-γ by the Type III Secretion System of *Yersinia pestis*

**DOI:** 10.1371/journal.pone.0015242

**Published:** 2010-12-13

**Authors:** Claire Gendrin, Stéphane Sarrazin, David Bonnaffé, Jean-Michel Jault, Hugues Lortat-Jacob, Andréa Dessen

**Affiliations:** 1 Institut de Biologie Structurale, UMR 5075 (Comissariat à l'Enérgie Atomique/Centre National de la Recherche Scientifique/Université Grenoble I), Grenoble, France; 2 Laboratoire de Chimie Organique Multifonctionnelle, Institut de Chimie Moléculaire et des Matériaux d'Orsay, UMR 8182, Université Paris-Sud 11, Orsay, France; Tulane University, United States of America

## Abstract

*Yersinia pestis*, the causative agent of bubonic plague, employs its type III secretion system to inject toxins into target cells, a crucial step in infection establishment. LcrV is an essential component of the T3SS of *Yersinia* spp, and is able to associate at the tip of the secretion needle and take part in the translocation of anti-host effector proteins into the eukaryotic cell cytoplasm. Upon cell contact, LcrV is also released into the surrounding medium where it has been shown to block the normal inflammatory response, although details of this mechanism have remained elusive. In this work, we reveal a key aspect of the immunomodulatory function of LcrV by showing that it interacts directly and with nanomolar affinity with the inflammatory cytokine IFNγ. In addition, we generate specific IFNγ mutants that show decreased interaction capabilities towards LcrV, enabling us to map the interaction region to two basic C-terminal clusters of IFNγ. Lastly, we show that the LcrV-IFNγ interaction can be disrupted by a number of inhibitors, some of which display nanomolar affinity. This study thus not only identifies novel potential inhibitors that could be developed for the control of Yersinia-induced infection, but also highlights the diversity of the strategies used by *Y. pestis* to evade the immune system, with the hijacking of pleiotropic cytokines being a long-range mechanism that potentially plays a key role in the severity of plague.

## Introduction

*Yersinia pestis* is the etiologic agent of plague, a devastating, acute infectious disease responsible for the death of 200 million people throughout the course of three worldwide pandemics [1]. The infectious mechanism of *Y. pestis* is strictly dependent on its type III secretion system (T3SS), a complex macromolecular structure present on the bacterial surface. The system serves as a conduit to inject T3SS-specific toxins directly into the cytosol of target cells. It is composed of over twenty macromolecules that associate into a basal structure spanning both bacterial membranes, and is terminated by a hollow needle through which toxins are believed to travel in semi-unfolded state [2]–[4]. Toxin entry into the eukaryotic cytoplasm requires the formation in the target membrane of a structure called ”translocon”, which is composed of two T3SS-encoded membrane proteins and one hydrophilic partner (the V antigen) [4], [ 5]–[7]. Similarities between proteins from microorganisms carrying T3SS within the same family can also be observed at the structural level [8]–[10]. However, secreted toxins are specific to each pathogen. In *Y. pestis*, genes that encode proteins required for the formation of the T3SS are present on a mobile genetic element (pCD1), which also carries genes for a set of secreted antihost proteins (Yops), as well as the V antigen (LcrV), all of which are absolutely necessary for infection [11]–[13].

Bacterial invasion of a mammalian host usually results in a prompt inflammatory response, generating the upregulation of major proinflammatory cytokines, which corresponds to the first level of innate immune defense. If this process on its own is not capable of destroying invading organisms, at least it allows the host to perform clonal selection and amplification of T-lymphocytes, mediators of specific immunity [12]. It has been shown that all three pathogenic *Yersinia* species (*Y. pestis* and the entheropathogenic species *Y. pseudotuberculosis* and *Y. enterocolitica*) are able to down-regulate inflammation mechanisms. In the case of *Y. pestis*, this down-regulation is strictly dependent on a functional T3SS [14], and most specifically, on the presence of the V-antigen LcrV. In addition to being detected in secreted form in the extracellular milieu upon activation of the T3SS, LcrV forms a distinct structure at the tip of the type III secretion needle and is associated to YscF, the needle-forming subunit [15], [16].

The numerous studies undertaken on LcrV from the various *Yersinia* species have shown that LcrV plays a major role at the interface with the immune system of the host, being the major protective antigen against the different forms of *Yersinia* infection [17], [18] and inducing the expression of the anti-inflammatory cytokine IL-10, which prevents upregulation of the pro-inflammatory cytokines TNF-α and IFNγ [19], [20]. IFNγ breakdown has been observed in the spleens of infected mice, and active immunization with an LcrV-derived fusion protein restores a normal level of synthesis [19]. IFNγ is a pleiotropic cytokine secreted by natural killer cells and T cells during innate immune processes. It interacts with a specific ubiquitous membrane receptor (IFNγR), which triggers expression of a variety of proteins [21]. The IFNγ chain is 143-amino acids long and is active as a homodimer; its structure reveals two intertwined polypeptides carrying globular N-terminal domains and a flexible C-terminal region [22]. Receptor recognition involves amino acids 18–26 and 108–124 of the N-terminal region [23], [24], as well as a segment of the C-terminal flexible tail (residues 125–134, 25). This C-terminal region includes residues KRKR_128–131_, which are also involved in binding to heparan-sulfates (HS), highly sulfated glycosaminoglycans present in the extracellular matrix and at the cell surface [26]. The competitive binding of IFNγ to HS allows regulation of the cytokine activity *in vivo* [25], [27].

Recently, it has been proposed that LcrV from *Y. pestis* exhibits IFNγ-dependent binding to specific monocytic cell lines [28]. In addition, inhibition of TNF-α synthesis and induction of IL-10 expression were observed as a consequence of this interaction. This study raised the question of a direct inhibition of IFNγ by LcrV, in addition to its role as an inhibitor of cytokine expression. We thus set out to characterize a potential LcrV-IFNγ complex using biophysical methods and investigate the potential for its disruption by small molecule inhibitors. In the present study, we demonstrate the formation of an LcrV-IFNγ complex, whose association occurs with nanomolar affinity even in the absence of IFNγR. By employing a panoply of IFNγ mutants, we mapped the LcrV-IFNγ interaction region and found that it overlaps the HS binding site of the cytokine. Based on this observation, we also demonstrate that HS-like inhibitors are capable of blocking this specific association. The molecules which demonstrate the highest affinity constitute interesting candidates for the development of novel antibacterials which could potentially interfere with *Yersinia*'s immune system subversion mechanism.

## Materials and Methods

### Protein expression and purification

Expression of GST-LcrV [29], of PcrV [6] and of IFNγ [30] was performed in the E. coli BL21 DE3 strain (T7 express, Biolabs) grown in 1 L of Luria broth medium. Expression was induced with 1 mM isopropyl 1-thio-β-D-galactopyranoside at an A_600_ of 0.7. For GST-LcrV and His-PcrV, growth was carried out for 3 hours at 37°C at 180 rpm. GST-LcrV expressing cells were harvested and lysed by sonication in buffer A (50 mM Tris-HCl, pH 7.5, 150 mM NaCl, 1 mM EDTA). The cell lysate was cleared by centrifugation and the supernatant was applied onto a GST-trap column (GE Healthcare) pre-equilibrated in buffer A. The protein was eluted with buffer A containing 10 mM of reduced glutathione. Digestion of the GST tag was performed with 50 units of PreScission protease™ (Amersham Pharmacia Biotech Inc) for 3 h at room temperature. The digestion products were reloaded onto the GST-column to allow separation of proteolyzed LcrV from the GST domain. Fractions containing LcrV were pooled and applied to a gel filtration column (Hiload 16/60 Superdex™200, GE Healthcare) equilibrated in 10 mM HEPES, pH 7.4, 150 mM NaCl, 1 mM EDTA (buffer B).

Cells expressing His-PcrV were also lysed by sonication in 25 mM Tris-HCl, pH 8.0, 200 mM NaCl, 2% glycerol. The cleared lysate was loaded onto a Ni-Sepharose column (GE Healthcare). PcrV was eluted with a step imidazole gradient and dialyzed to remove imidazole. Thrombin digestion allowed cleavage of the His tag, which was retained on the Ni-column upon reloading of the cleaved material. His-tag free PcrV was then submitted to gel filtration using a Hiload 16/60 Superdex™200 column (GE Healthcare) in Tris 25 mM, pH 8.0, 100 mM NaCl, 1 mM EDTA.

IFNγ-expressing cells were induced 5 h at 37°C, and lysed by two passages through a French press in lysis buffer (50 mM Tris, pH 7.2, NaCl 100 mM) Purification from inclusion bodies was performed as previously described [31] with slight modifications. Briefly, inclusion bodies were solubilized in 6 M guanidine HCl, and the protein was refolded in a 12.5 volume of phosphate buffer, pH 7.5. IFNγ was purified by ion exchange on a home-packed 5 mL source S column (resin bought from GE Healthcare) and gel filtration (Hiload 16/60 Superdex™200, GE Healthcare) in buffer B. The integrity of all three purified proteins was checked by mass spectrometry.

### IFNγ mutants

Mutagenesis was performed using the QuickChange II Site-Directed Mutagenesis Kit (Stratagene) following manufacturer's instructions. The vector pET11a containing the wild-type human IFNγ cDNA was used as the DNA template. Complementary primers were designed as to convert the selected basic amino acid into a serine residue. Amino acids were mutated one by one, each mutated vector being used as a template for the following mutation.

### Biacore-based binding assays

Binding analyses were performed using a Biacore 3000 system. Flow cells of a CM3 sensor chip were activated with 0.2 M *N*-ethyl-*N*'-(diethylaminopropyl)-carbodiimide and 0.05 M *N*-hydroxysuccinimide (EDC/NHS). The flow cell destined to recieve LcrV was then submitted to the injection of 80 mM 2-2-(pyridinyldithio)ethaneaminehydrochloride (PDEA thiol coupling reagent) in 0.1 M borate buffer, pH 8.5, which allows quantitative activation of NHS esters on the surface. Following PDEA injection, 35 µL of LcrV at 50 µg/mL in 10 mM sodium acetate buffer, pH 4.1, were injected, which typically allowed coupling of 1200–2000 resonance units (RU) of the protein on the surface. Excess reactive groups were deactivated by injecting 50 mM L-cysteine in formate buffer, pH 4.3, containing 1 M NaCl. For comparison of LcrV and PcrV properties, proteins were bound directly after activation by injecting 50 µL of the purified protein at the concentration of 100 µg/mL. The remaining activated groups were blocked with 1 M ethanolamine, pH 8.5. For all experiments, a control flow cell was prepared by injection of 10 mM sodium acetate buffer, pH 4.1, directly after activation, followed by blocking with 1 M ethanolamine, pH 8.5. For IFNγR binding experiments, a flow cell of a CM4 sensor chip was EDC/NHS activated as described above, after which 50 µL of Streptavidin (Sigma-Aldrich) at 200 µg/mL in 10 mM sodium acetate buffer, pH 4.1, were injected. Biotinylated IFNγR (0.3 mg/mL) was then captured to a level of 1600 RU on this surface.

For binding assays, IFNγ and its derivatives were simultaneously injected over the surface presenting the tested protein and the negative control. Surfaces were regenerated using 12 µL pulses of 10 mM HCl. To determine the equilibrium constant (Kd), the binding responses at the end of the injection phase were extracted from the sensorgrams, and plotted according to the Scatchard representation (R_eq_/C against R_eq_, where R_eq_ is the steady state value at equilibrium and C the concentration of the injected cytokine. To test the potential inhibitors of the LcrV-IFNγ interaction, 0.5 equivalents of each inhibitor were incubated with a 120 nM solution of IFNγ. The mix was injected over immobilized LcrV, and the percentage of inhibition was calculated at the end of the association phase. The inhibitory potential of 2O_32_ was further investigated by preincubating increasing concentrations of 2O_32_ (0–350 nM) with IFNγ (120 nM), and injecting the complexes over immobilized LcrV. Fitting of the data was performed using the Origin 8.1 software (OriginLab). During Biacore studies, the chips were submitted to continuous flow of HBS-P buffer (10 mM Hepes, 0.15 M NaCl, and 0.05% P20 detergent, pH 7.4), and all injected material were diluted in the same buffer. Except when indicated, reagents used for these studies are from GE Healthcare.

### Chemical modification by N-Bromosuccinimide (NBS)

A 10 mM solution of LcrV was prepared in 50 mM Tris-acetate, pH 5.0, 20% glycerol, 1 mM EDTA. N-Bromosuccinimide (NBS, Sigma) was resuspended in the same buffer and an 80-fold molar excess was added to the LcrV solution. After a 10 min incubation at 30°C in the dark, unreacted NBS was neutralized by an equal amount of free L-tryptophan, and precipitated material was eliminated by centrifugation. Modified LcrV was equilibrated in buffer B by gel filtration in PD-10 desalting columns (GE Healthcare).

### Intrinsic fluorescence measurements

Tryptophan fluorescence emission spectra were measured on a PTI quanta master 4 (Photon Technology International, London, ON, Canada) at 25°C. Emission spectra were recorded from 310 to 370 nm using an excitation wavelength of 295 nm, with a 2 nm excitation and a 4 nm emission band pass. The cuvette contained 1 mL of 1 µM IFNγ, and increasing concentrations of oxidized LcrV were added. The values were corrected for dilution and for LcrVox inner-filter effect using another cuvette containing 4 µM of N-acetyltryptophanamide (NATA), as previously described [32]. Data were analyzed using the GraFit Erithacus 5.0 software. The experimental data were fit to the equation:
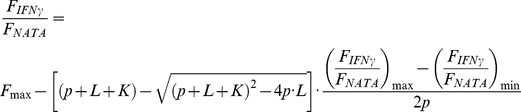
where *F* corresponds to the intensities of the various spectra analyzed, *p* is the concentration of IFNγ in the cuvette, *L* is the concentration of injected LcrVox, and *K* is the dissociation constant.

## Results

### LcrV interacts directly with IFNγ

**Figure 1:**
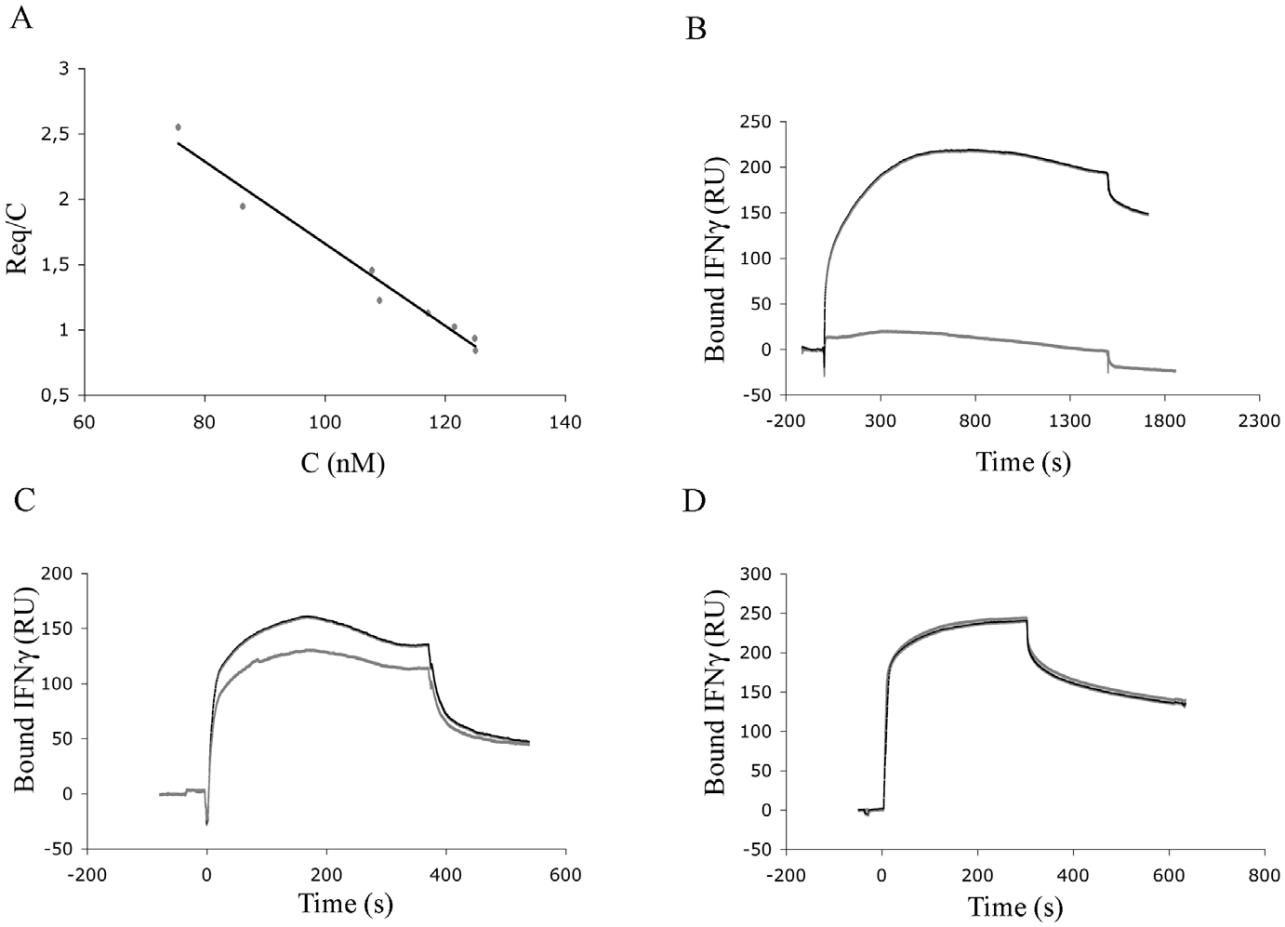
Free IFNγ interacts with LcrV, but not with PcrV, in a surface plasmon resonance assay. (A) Scatchard analysis of IFNγ binding to LcrV immobilized on a CM3 sensorchip (1150 RU). R_eq_, steady state value at equilibrium; C, concentration of injected IFNγ. (B) 5.0 µg/mL IFNγ was injected over LcrV (2700 RU, black curve) and PcrV (1200 RU, grey curve) immobilized on two different lanes of the same sensorchip. (C) Injection over immobilized LcrV (1150 RU) of 2 µg/mL IFNγ (black curve) or of 2 µg/mL IFNγ and an equimolar amount of IFNγR (grey curve). RU =  resonance units. (D) Injection of 1 µg/mL IFNγ alone (black curve) or in combination with LcrV (1 µg/mL of each, grey curve) over immobilized IFNγR (1600 RU). For all experiments, non-specific binding to the sensor chip was subtracted from the raw data.

LcrV was shown to bind to human U937 monocytic leukemia cells and human alveolar macrophages, provided that human IFNγ was present; in addition, it was suggested that cellular recognition occurred through interaction of LcrV with the IFNγR-IFNγ complex [28]. In order to further characterize the immuno-regulatory function of LcrV, we explored the LcrV-IFNγ interaction (in the presence or absence of IFNγR) using a Biacore-based approach. We took advantage of the fact that LcrV possesses a single cysteine residue to immobilize it in an oriented fashion on a CM3 sensorchip, as described in the methods section. Injection of IFNγ over the surface produced a significant binding that demonstrated the existence of an IFNγ-LcrV complex (Figure 1A) IFNγ was found to bind immobilized LcrV in a concentration-dependent manner, and a Scatchard analysis of the binding responses obtained at equilibrium produced a dissociation constant (K_d_) of 32 nM (Figure 1A). PcrV, the LcrV homolog from *P. aeruginosa*, was also immobilized on a CM3 sensorchip and tested for its ability to bind IFNγ (Figure 1B). Notably, almost no binding was observed even at the highest concentration of 5 µg/mL IFNγ.

LcrV was also tested for its ability to interact with IFNγR-IFNγ (Figure 1C and 1D). Interestingly, the signal obtained upon injection of the complex IFNγ-IFNγR was of lower value than that observed when we injected the same concentration of purified IFNγ, which suggests that LcrV may not bind to the IFNγR-IFNγ complex as well as to IFNγ alone. Subsequently, biotinylated IFNγR was immobilized on a streptavidin-coated surface, and IFNγ alone, or a mixture of LcrV and IFNγ, were injected over the surface (Figure 1D). The binding of IFNγ to its receptor was not modified in the presence of LcrV, which strongly suggests that the interaction between LcrV and IFNγ does not prevent the association of IFNγ with its receptor. Thus, these data indicate that LcrV binds IFNγ with nanomolar affinity, even in the absence of the IFNγ receptor.

### Fluorescence analysis of the LcrV-IFNγ complex

**Figure 2:**
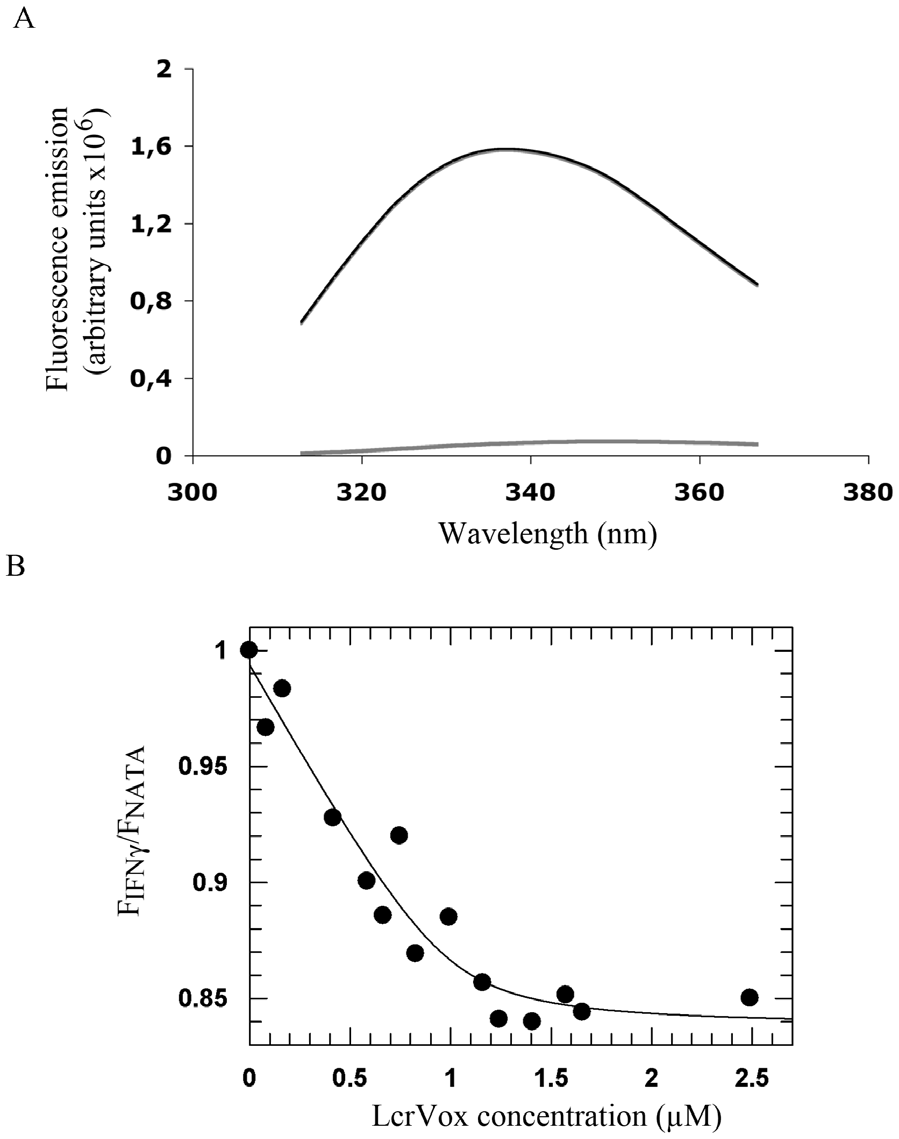
Characterization of the LcrV/IFNγ interaction by intrinsic fluorescence studies. (A) Effects of NBS modification on LcrV spectral properties. LcrV was incubated with an 80-fold molar excess of NBS, and the resulting fluorescence (grey curve) was compared to that of wild-type LcrV (black curve). The fluorescence emission spectrum was recorded over the range 310–370 nm after excitation at 295 nm. (B) Evolution of the relative fluorescence intensities over injection of increasing concentrations of oxidized LcrV (LcrVox). Fitting of the data was performed as described in the experimental section. The LcrV curve was not shown for clarity. F_IFNγ_; intensity of the IFNγ spectrum; F_NATA_, intensity of the NATA spectrum.

In order to further characterize the interaction between LcrV and IFNγ, we measured the change of intrinsic fluorescence of IFNγ upon complex formation. LcrV and IFNγ each include one single tryptophan residue, whose spectral property could potentially be altered upon their interaction. To precisely dissect the fluorescence events related to complex formation and pinpoint uniquely the contribution of the tryptophan found in IFNγ, we treated LcrV with N-Bromosuccinimide (NBS) [32], in order to chemically modify its Trp 113. This chemical oxidation produced drastic changes on the LcrV emission spectrum, characterized by a large decrease in intensity (Figure 2A).

Oxidized LcrV (LcrVox) was then introduced into a cuvette containing 1 µM IFNγ, and the variations of IFNγ emission spectra were recorded between 310 and 370 nm. Control injections of LcrVox into a solution of N-acetyltryptophanamide (NATA) allowed correction of the inner-filter effect of LcrVox. The fitting of the data enabled the determination of a Kd value of 42 nM (Figure 2B), which is comparable to that obtained from the Biacore studies (see above). These data lead to the conclusion that, whether LcrV is immobilized or free in solution, it binds to IFNγ with strong affinity.

### The two basic clusters of the IFNγ C-terminal domain are crucial for the interaction with LcrV

**Figure 3:**
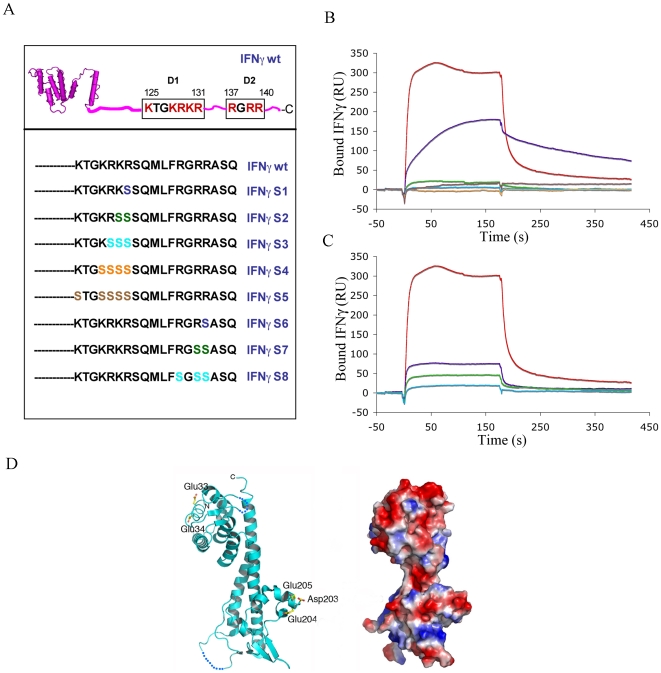
Binding of wild-type IFNγ and its mutants to LcrV demonstrated by surface plasmon resonance. (A) Presentation of the IFNγ mutants used in this study. Serial substitutions of R and K by S residues allow specific impairment of the two basic clusters of the Ct domain of IFNγ. IFNγwt: wild-type IFNγ. (B) Injection over immobilized LcrV (1150 RU) of IFNγwt (red curve), mutants IFNγS1 (blue), IFNγS2 (green), IFNγS3 (light blue), IFNγS4 (orange) and IFNγS5 (brown). (C) Injection of wild-type IFNγ (red curve), IFNγS6 (blue), IFNγS7 (green), and IFNγS8 (light blue) over the same sample of LcrV. For (B) and (C), a concentration of 4 µg/mL of each protein was injected at 20 µL/min. Non-specific binding to the sensor chip was subtracted from the raw data. (D) The surface of LcrV is highly acidic. Left: ribbon diagram indicating the two acidic patches suggested by Abramov *et al.* [28] as playing a role in IFNγ recognition. Right: Surface electrostatics of LcrV in the same orientation. Acidic regions are in red, basic in blue.

IFNγ is a dimeric cytokine whose biological activity is related to its unstructured C-terminal domain [reviewed in 21]. This domain includes two basic patches, D1 and D2 (amino acids 125–131 and 137–140, respectively, see Figure 3A), both of which have been reported to bind HS [33]. Notably, the basic clusters of IFNγ (and specifically the GRRA_138–141_ motif, which overlaps the second basic cluster) play a role in LcrV recognition in cell culture conditions [28].

In order to precisely map the LcrV-IFNγ interaction region, we investigated whether the two basic clusters of IFNγ were involved in the interaction with immobilized LcrV by surface plasmon resonance. We generated specific mutants of IFNγ which display serial mutations of R and K residues either within the D1 or D2 domain, allowing an independent assessment of the role of each of these specific basic clusters (Figure 3A). When R^131^ of cluster D1 was substituted by a serine residue, the sensorgram reached a maximum RU value of 175, compared to 300 RU for wild-type IFNγ. Further mutations within the D1 domain (mutants S2 to S5, Figure 3B) strongly impaired binding, demonstrating the importance of cluster D1 for the interaction to take place. An even more pronounced phenomenon was observed when IFNγ basic cluster D2 was modified, with a single mutation at position 140 almost completely abolishing LcrV recognition. Binding to immobilized LcrV decreased gradually for mutants S7 and S8, as compared to wild-type IFNγ. Our data thus indicate that both basic clusters of IFNγ are fundamental for the interaction with LcrV, with a strong involvement of the most distal one (D2). Notably, the surface of LcrV is highly acidic (Figure 3D, see below), providing a number of possibilities for recognition of the basic patches on IFNγ.

Interestingly, however, a peptide corresponding to the C-terminal region of IFNγ encompassing both clusters D1 and D2 could not be shown to interact with LcrV neither by Biacore nor fluorescence techniques (not shown), suggesting that this region must be stabilized or properly oriented within the full-length IFNγ structure in order to be available for productive binding with partner molecules.

### Inhibition of formation of the LcrV-IFNγ complex

**Figure 4:**
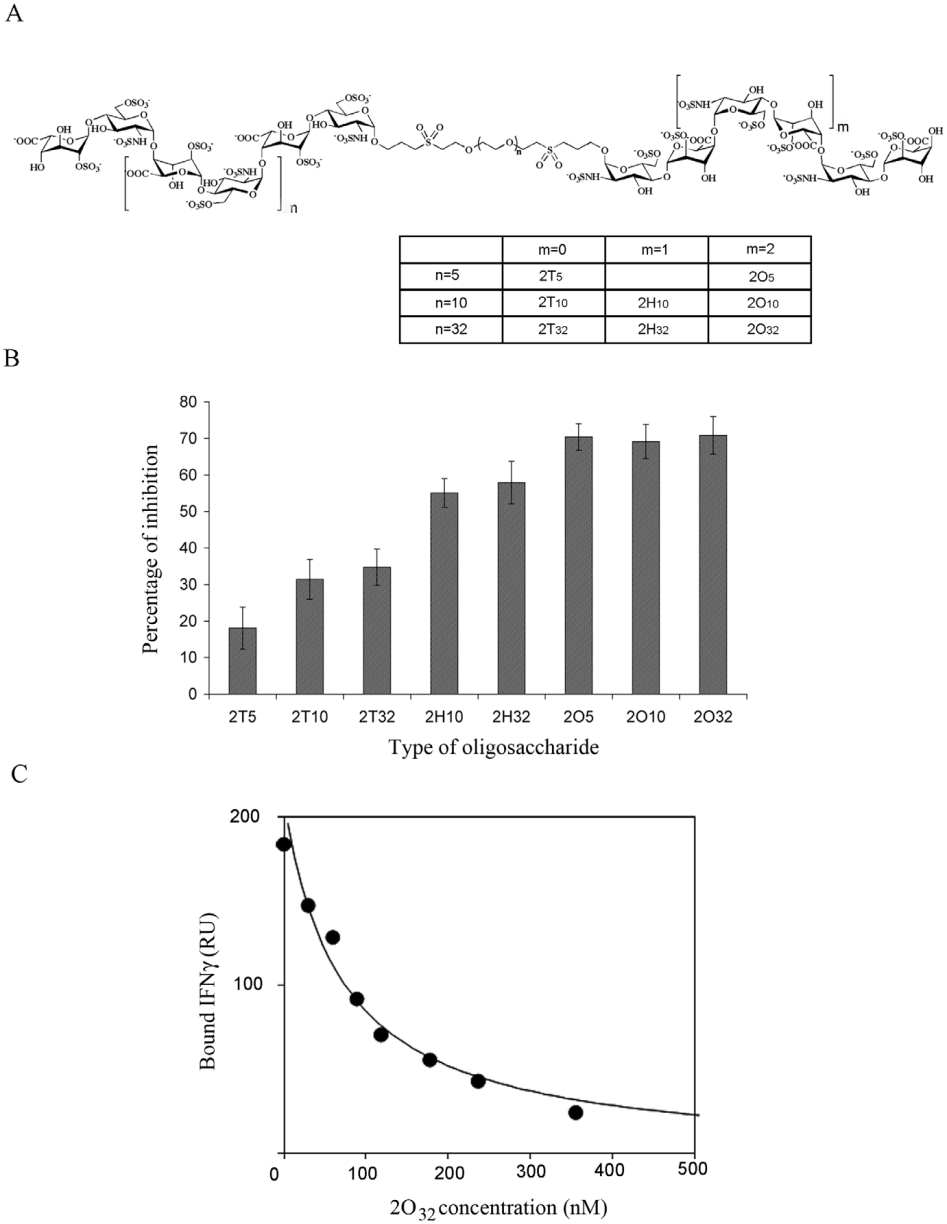
Inhibition of LcrV/IFNγ interaction by different synthetic oligosaccharides. (A) Formula of the different oligosaccharides used in this study. m, number of disaccharide repeats, n = number of ethylene glycol repeats. Adapted from ref 51. (B) Each inhibitor tested was incubated with IFNγ, and complexes were injected over immobilized LcrV (2000 RU) at 10 µL/min. Non-specific binding to the sensor chip was subtracted for each injection. The percentage of inhibition is represented for three independent experiments, and the standard error of the mean is indicated (bars). The mean response in the absence of inhibitor (0% of inhibition) was equal to 270 RU. (C) IFNγ was pre-incubated with a range of concentrations of 2O_32_. Each reaction mixture was injected over immobilized LcrV (1150 RU) at 10 µL/min. Non-specific binding to the sensor chip was subtracted for each injection.

A set of synthetic glycoconjugates that mimic HS were synthesized and tested for their ability to interact with IFNγ [30]. These molecules were shown to inhibit the binding of IFNγ to heparin, the most active one displaying an IC_50_ value of 35–40 nM. Since the formation of the IFNγ-HS and LcrV-IFNγ complexes share a common dependency on the basic residues present in the C-terminus of IFNγ, these data prompted us to test whether these synthetic glycoconjugates would also inhibit the IFNγ-LcrV interaction in a Biacore study. We thus chose a set of compounds, each composed of two tetrasaccharides (T), hexasaccharides (H) or octasaccharides (O), which were linked to poly(ethylene glycol)-based spacers of different lengths (Figure 4A). Glycoconjugates were incubated with IFNγ in a 0.5∶1 molar ratio, and the complexes were injected over immobilized LcrV. The binding obtained at equilibrium was compared to that observed for free IFNγ, which was considered as 0% inhibition. Results from three independent experiments indicated that all tested compounds inhibited LcrV-IFNγ assembly (Figure 4B), with the octasaccharides being the best inhibitors (approx. 70% of inhibition).

We further characterized the inhibition properties of 2O_32_, chosen as the most potent inhibitor. Increasing ratios of 2O_32_:IFNγ were injected over immobilized LcrV, and the binding responses were analyzed as described above. The inhibition curve of 2O_32_ is shown in Figure 4C. Fitting of the data leads to an IC_50_ of 75 nM, which is comparable to the IC_50_ value of 35–40 nM obtained for the inhibition of IFNγ-heparin binding by 2O_10_ [30]. These data thus allowed the identification of efficient inhibitors of the LcrV-IFNγ interaction, which could constitute potential candidates for preventing *Yersinia* dissemination over the course of infection.

## Discussion

The type III secretion system is a complex macromolecular system employed by a number of human pathogens to inject toxins directly into the cytoplasm of target eukaryotic cells and thus initiate infection [4]. LcrV is secreted by the T3SS and is a major regulator and effector of virulence [12], [34]. Passive protection against *Y. pestis* was observed in mice using anti-V antibodies that recognize one or more internal epitopes located between amino acids 168 and 275, which underlines the highly protective antigenic character of LcrV [17]. LcrV interactions with the immune system also include an anti-inflammatory role: LcrV is able to trigger secretion of IL-10, a powerful anti-inflammatory cytokine that prevents expression of different host inflammatory factors [35], [36]. This induction of secretion could be partly explained by the binding of LcrV to receptor-bound IFNγ [28].

The results presented here provide evidence that LcrV is able to interact with free IFNγ even in the absence of IFNγR. Addition of IFNγR to IFNγ did not improve binding, but in contrast partially prevented optimal interaction of IFNγ with immobilized LcrV. IFNγ residues involved in receptor binding (amino acids 18–26 and 108–124) are located in the globular structured part of the molecule [23], [24], and it has been shown that the C-terminal domain of IFNγ is partially buried within the IFNγ-IFNγR complex [24], [25]. Our results demonstrate that LcrV recognizes the unstructured flexible C-terminal domain of IFNγ (residues 124–143). These elements provide an explanation for the decreased binding of IFNγ to LcrV in the presence of IFNγR: titration of IFNγ by its receptor causes steric hindrance for LcrV recognition, thus blocking the accessibility of the IFNγ C-terminus for interaction with LcrV. Notably, Abramov and co-workers [28], by using flow cytometry, reported that LcrV recognizes IFNγ when it is in complex with its receptor (IFNγR). Here, we employ different biochemical and biophysical techniques to show that IFNγR is not required for LcrV recognition of IFNγ, thus underlining its potential as a regulator of inflammation.

In addition, we show by surface plasmon resonance that the interaction of IFNγ with LcrV mostly relies on D1 and D2, the two basic clusters present in the C-terminus of the protein. Basic residues in both clusters were sequentially disrupted, which showed that D2 was slightly more important than D1 for the interaction. These results are in accordance with previous observations made by Abramov *et al.*, who showed that a truncated form of IFNγ lacking cluster D2, and mouse IFNγ, which includes only cluster D1, are unable to interact with LcrV when added to U937 cells or alveolar macrophages. Additionally, our results point out the prominent role of cluster D1 in the strong affinity of IFNγ for LcrV.

PcrV is the homologue of LcrV in the well-studied *Pseudomonas aeruginosa* TTS system [37]–[41]. Both proteins share 41% sequence identity and their role in translocon assembly and toxin translocation have been proposed to be similar [16], [29]. However, when PcrV was tested for direct binding to IFNγ by surface plasmon resonance, no interaction was observed. LcrV contains a number of acidic residues including LEEL_32–35_ and DEEI_203–206_ motifs that have been proposed as being involved in IFNγ binding through electrostatic interactions with the C-terminal positive charges of IFNγ [28]. The crystal structure of LcrV, which shows that the molecule is shaped like a dumbbell with both N- and C-termini on the same side (Figure 3D) [42], indicates that both motifs are exposed on the protein surface, thus being potentially available for interactions with other protein partners (Figure 3D). The surface of LcrV, however, is highly acidic, as indicated by the elevated number of exposed negative charges (shown in red in Figure 3D), and thus the participation of additional acidic patches in the interaction with IFNγ cannot be ruled out. Interestingly, the sequence of PcrV displays a region with sequence similarity to the LEEL_32–35_ motif of LcrV (residues QEEL_29–32_), but lacks an acidic region which could correspond to LcrV's DEEI_203–206_ motif. The absence of this specific surface acidic patch could explain why PcrV does not interact with IFNγ in spite of a high degree of homology with LcrV. Of note, it has been shown that recombinant LcrV and PcrV also differ in their ability to induce IL-10 or suppress TNF-α production by stimulated macrophages [43]: while LcrV elicits an extended immunosuppressive response, PcrV's interactions with the immune system may be limited to its immunogenic character [44], [45]. These elements are particularly interesting when considered in light of the respective degree of virulence of both pathogens: plague bacilli must kill their host to survive in nature [12], which may require a rapid and global neutralization of the host immune system, whereas *P. aeruginosa* is considered as an opportunistic microorganism, able to develop a chronic infection that will survive the immune response.

The relevance of the IFNγ-LcrV interaction *in vivo* remains to be investigated. It has been shown that LcrV displays differential targeting profile in the host: it is located on the extracellular surface of the bacterium [46], [47], where it may act as an assembly platform for the translocation pore [15], [29], [48], but it is also released from *Yersinia* into the extracellular medium [49], [50]. The latter authors have also reported that a pool of LcrV may be injected into the host cell cytoplasm, where it could interfere with cellular processes required to eliminate *Yersinia* during infection. A global model of LcrV targeting was proposed, which suggests its dynamic association at the bacterial surface, allowing partial release in the surrounding medium [50]. This model is consistent with an immunomodulatory role for LcrV: released LcrV would be free to diffuse away and have a broadly local or even a systemic effect during an infection process. We propose that this pool of secreted LcrV is involved in the interaction with IFNγ, acting as a long-distance weapon to prevent the multiple effects of the cytokine.

In light of this model, it appears crucial to develop new molecules that could interfere with the extended anti-immune properties of LcrV. The oligosaccharides used in this study constitute particularly interesting candidates as they inhibit the interaction between LcrV and IFNγ with a high efficiency. Further *ex vivo* and *in vivo* studies will be needed to test their anti-*Yersinia* potential, but several factors are encouraging: i) their small size allows an efficient synthesis of homogenous preparations, ii) they are heparin-like, and thus presumably non-immunogenic compounds, and as such they should be well tolerated by the organism, iii) they were specifically designed to interact with IFNγ [51], and they represent interesting scaffold structures for further developments.

Cytokine network perturbation is one of the strategies exhibited by pathogenic bacteria to counteract the host immune system [52]–[54]. The proteins responsible for these effects have been named “bacteriokines” [53]. LcrV can be classified into this group since i) a protein A-LcrV fusion induces suppression of TNF-α and IFNγ in spleen homogenates of mice, which promotes *in vivo* survival of the bacteria [14], [19], ii) a polyhistidine fusion of LcrV causes an increase of IL-10 expression at the spleen level [20], and iii) in the present study, we show that LcrV also directly interacts with IFNγ *in vitro*. This additive anti-inflammatory mechanism probably enables the invading bacteria to achieve a lethal cellular burden before an effective specific immune response can be initiated. Therefore, the identification of specific inhibitors of this interaction, provided in this study, is of major importance and provides interesting potential candidates for the development of new antibiotics against *Yersinia* spp.
